# Expression and intracellular localization of duck enteritis virus pUL38 protein

**DOI:** 10.1186/1743-422X-7-162

**Published:** 2010-07-17

**Authors:** Jun Xiang, Guangpeng Ma, Shunchuan Zhang, Anchun Cheng, Mingshu Wang, Dekang Zhu, Renyong Jia, Qihui Luo, Zhengli Chen, Xiaoyue Chen

**Affiliations:** 1 Avian Diseases Research Center, College of Veterinary Medicine of Sichuan Agricultural University, Yaan, Sichuan 625014, China; 2 China Rural Technology Development Center, Beijing, 100045, China; 3 Key Laboratory of Animal Diseases and Human Health of Sichuan Province, Yaan, Sichuan 625014, China; 4 Epizootic Diseases Institute of Sichuan Agricultural University, Ya'an, Sichuan 625014, China

## Abstract

Knowledge of the intracellular location of a protein can provide useful insights into its function. Bioinformatic studies have predicted that the DEV pUL38 mainly targets the cytoplasm and nucleus. In this study, we obtained anti-pUL38 polyclonal sera. These antibodies were functional in western blotting and immunofluorescence in DEV-infected duck embryo fibroblasts (DEFs). pUL38 was expressed as a 51-kDa protein from 8 h post-infection onward, initially showing a diffuse distribution throughout the cytoplasm, and later in the nucleus. Furthermore, pUL38 was found in purified virus. These results provide the first evidence of the kinetics of expression and intracellular localization of DEV pUL38.

## Findings

Duck enteritis virus (DEV) is a natural pathogen of ducks and causes duck viral enteritis, an acute, contagious, and lethal disease affecting waterfowl belonging to the family Anatidae [1]. DEV is a member of the family *Herpesviridae*. The DEV virion is enveloped, and the genome consists of double-stranded DNA segments packaged in an icosahedral capsid [2]. The gene library of the DEV CHv strain was constructed in our laboratory, and more than 72 major open reading frames (ORFs) were found [3], coding for enzymes, structural proteins, and scaffolding proteins. However, the functional characteristics of most of these proteins are still unknown. To date, only the kinetics of expression and intracellular location of pUL24 [4], pUL31 [5,6], pUL51 [7,8], pUS3 [9], and dUTPase [10] have been investigated. Using bioinformatic tools, some putative glycoproteins and enzymes of the virus were characterized, such as gC [11], gE [12], gI, gD [13], and helicase pUL5 [14]. The identity of other components remains obscure. The DEV pUL38 protein has been suggested to be a putative structural protein. Computational predictions have revealed that DEV pUL38 mainly targets the cytoplasm and nucleus [15]. Immunological assays are an essential part of studies aimed at determining the kinetics of expression and the cellular location of DEV pUL38 in vitro. In this study, we obtained rabbit anti-pUL38 polyclonal sera, which were shown to be functional in immunofluorescence and western blotting assays.

The DEV CHv strain used throughout this study was grown in duck embryo fibroblast (DEF) cells. Cell cultures were maintained in modified Eagle's medium (MEM) supplemented with 10% fetal bovine serum (FBS) and antibiotics [16]. In a previous study, we had amplified the ORF of pUL38 (1398 bp) from the DEV genome [15]. The amplified product was cloned between the *Bam*HI and *Xho*I sites of a pET32(+) plasmid, and a pET32-pUL38 plasmid construct was created.

**Figure 1:**
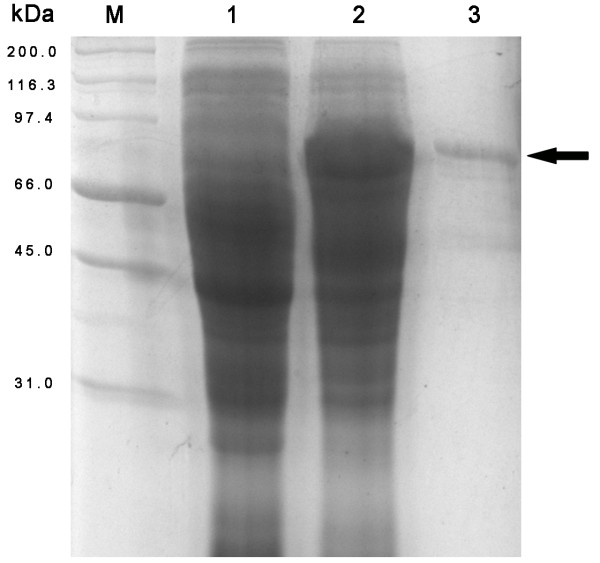
**Expression and purification of the DEV pUL38**. SDS-PAGE of the expressed peptide in E. coli BL21 (DE3) is shown. *M *Marker; *1 *the total cell proteins uninduced with IPTG; *2 *the total cell proteins induced with IPTG; *3 *the insoluble fraction after purification with IMAC. The black arrow points to the recombinant pUL38 (approximately 70 kDa).

*Escherichia coli *BL21(DE3) was transformed with the recombinant construct, and protein expression was induced with 1 mM IPTG at 37°C for 4 h. The bacterial proteins were analyzed by 12% SDS-PAGE under denaturing conditions. Protein bands were visualized after staining with 0.1% Coomassie blue R250, and the protein concentration was determined using the software program BandScan 5.0 [17]. The recombinant pUL38 was successfully expressed in the transformed cells (Fig. 1).

The expressed recombinant pUL38, however, was trapped in inclusion bodies. The cells were harvested by centrifugation and resuspended in 20 mM Tris buffer (pH 8.0). The cells were later lysed by using lysozyme (0.1 mg/mL) at 4°C for 1 h and sonicated on ice for 5 min at an amplitude of 30% with a 30-s pulse frequency. The lysate was centrifuged at 10,000 × *g *for 20 min at 4°C. The pellet was washed twice with 2 M urea containing 50 mM Tris buffer (pH 8.0), 1 mM EDTA, 150 mM NaCl, and 0.1% Triton X-100. The suspension was centrifuged at 10,000 × *g *for 20 min at 4°C, and then the resulting precipitate was resuspended in regeneration buffer containing 6 M urea, 0.5 M NaCl, 20 mM Tris-HCl (pH 7.9) and incubated at room temperature (25°C) for 30 min. The incubated mixture was then centrifuged at 10,000 × *g *for 20 min. To further purify the proteins, the supernatant was then poured onto a purification column and allowed to bind for 1 h with gentle shaking. The recombinant His-tagged proteins were purified from the above supernatant by immobilized metal affinity chromatography (IMAC) on a Ni-NTA affinity resin (Bio-Rad, California, USA) according to the protocol of Cai et al. [18]. Finally, homogeneity of the proteins was verified by an SDS-PAGE assay (Fig. 1).

Preimmune serum was collected. New Zealand white rabbits were first immunized intradermally with a mixture of 1 mg purified recombinant pUL38 protein and an equal amount of complete Freund's adjuvant (Sigma, Shanghai, China). After 2 weeks, the rabbits were boosted twice subcutaneously with the same amount of recombinant pUL38 protein and an equal amount of incomplete Freund's adjuvant at a 1-week interval. Two weeks after the last immunization, the antiserum was harvested from the carotid artery.

**Figure 2:**
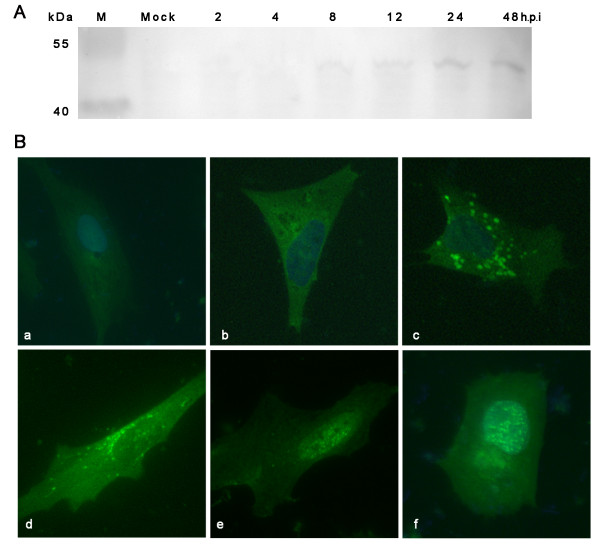
**Kinetics of expression and immunolocalization of the DEV pUL38 in infected DEF cells**. **A **Western blot of lysates from mock-infected or DEV-infected DEF cells with polyclonal antibodies specific to pUL38 protein, showing that pUL38 is expressed as a 51 kDa protein from 8 h onward following infection. **B **Immunofluorescence detection of pUL38 in mock-infected (a) or DEV-infected DEF cells at 8(c), 18(d), 30(e) and 56 h(f) post-infection. Cells were incubated with preimmune serum(b) pUL38-specific antibody and subsequently stained with fluorescein isothiocyanate (FITC)-conjugated secondary antibody. Nuclei were counterstained with DAPI (blue).

To determine the kinetics of pUL38 expression, DEF cells were infected with DEV. Cell lysates were prepared at 2 h, 4 h, 8 h, 12 h, 24 h, and 48 h post-infection (h.p.i). The DEV pUL38 protein was detected using rabbit polyclonal antibodies specific to the pUL38 protein. As seen in Fig. 2A, DEV pUL38 (molecular mass, approximately 51 kDa), was detectable in DEF cells as early as 8 h.p.i. The pUL38 expression increased over time and reached a maximum at approximately 48 h.p.i; this finding indicated that pUL38 is expressed throughout the viral replication cycle. The expression of *Herpesviridae *genes is temporally controlled and coordinated in a cascade fashion [19]. Immediate-early (α) transcripts are expressed first, and the proteins encoded by these mRNA species are required for the subsequent expression of all other kinetic classes of viral genes. Delayed-early (β) genes, many of which encode proteins involved in the replication of the viral genome, are maximally expressed before or at the peak of DNA replication and are then switched off. Late transcripts (γ) are maximally expressed only after the onset of viral DNA replication and encode proteins involved in virion assembly. As reported in previous studies, 2 identified immediate-early products, namely, protein kinase pUS3 and dUTPase, were first detected at 2 h.p.i. and 4 h p.i. respectively [9,10]. In contrast, 2 identified late products--tegument protein pUL31 and pUL51--were first detected at 6 h.p.i. and 8 h.p.i., respectively [5,8]. Hence, we concluded that pUL38 may be a late gene product and may be a part of virion architecture.

To confirm the intracellular distribution of pUL38, DEF cells were plated on coverslips and infected with DEV at an MOI of 5. The cells were processed at 8 h, 18 h, 30 h, and 56 h.p.i., and pUL38 was detected using pUL38-specific antibody and fluorescein isothiocyanate (FITC)-conjugated secondary antibody. As can be seen in Fig. 2B, the pUL38 distribution pattern appeared to change over the course of DEV infection. At 8 h.p.i., pUL38 was expressed diffusely throughout the cytoplasm of cells. At 18 h.p.i., it was detected close to the nucleus and showed a fine speckled pattern. At later times following infection (30 h), the pUL38 protein was localized in very fine punctate forms dispersed throughout the nucleus of infected cells. These results suggest a putative change in the intracellular localization of pUL38 during the course of DEV infection.

**Figure 3:**
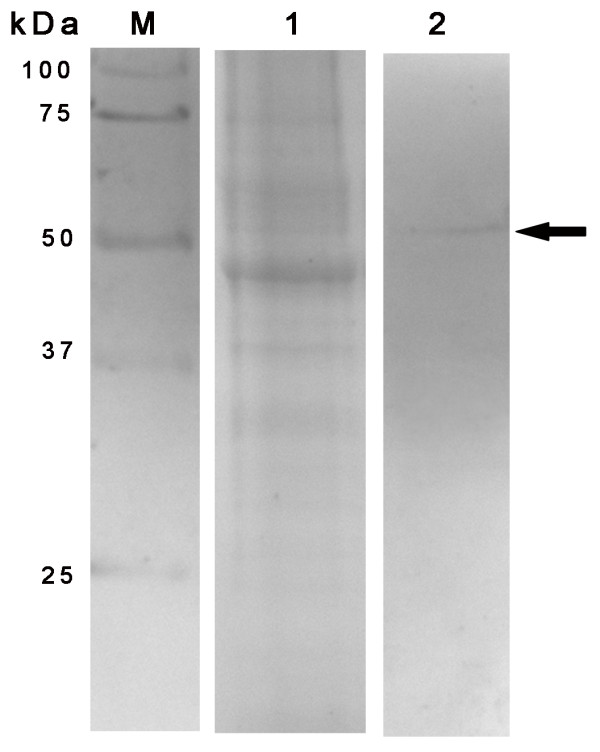
**The association of DEV pUL38 with purified virions**. Virus particles were collected from culture medium harvested at 48 h.p.i. and purified. Purified virions were lysed in SDS sample buffer, separated by SDS-PAGE, stained with Coomassie brilliant blue (lane 1), and then analyzed by western blotting with the UL38 antiserum (lane 2). Molecular mass marker sizes are shown on the left.

Since pUL38 is localized to the nucleus, we investigated the possibility of this protein being incorporated into DEV virions by probing the western blots of highly purified virions. The extracellular virions were collected from culture media harvested at 48 h.p.i. Virus particles were purified by sucrose density-gradient centrifugation [2]. The purified DEV virions were separated by SDS-PAGE, and western blots were performed with rabbit antisera against the pUL38 protein. A protein band corresponding to the molecular weight of 51 kDa was clearly seen in the blots (Fig. 3). This result suggests that pUL38 is a component of DEV virions.

In most herpesviruses, after assembly of the capsid and packaging of the viral genome--a process that occurs in the nucleus--the nucleocapsid is translocated to the cytoplasm [20]. For final maturation within the cytoplasmic tegument, components associate with the translocated nucleocapsid, with themselves, and with the future envelope; this results in the formation of an infectious herpes virion. However, there are 2 assembly pathways in DEV infection in both the cytoplasm and the nucleus [21]. The majority of nucleocapsids acquire teguments in the nucleus, which are enveloped by the inner nuclear membrane, after which mature viruses are released into the cytoplasm. However, there are some nucleocapsids that first assemble autocatalytically in the cytoplasm, and then acquire the cytoplasm tegument components. At later times following infection, pUL38 localized in the nucleus of infected cells and was not detectable in the cytoplasm. The results suggested that pUL38 may be an internal component of the DEV nucleocapsid and may be involved in stabilizing the capsid.

## Competing interests

The authors declare that they have no competing interests.

## Authors' contributions

JX, GPM and SCZ carried out most of the experiments and drafted the manuscript. ACC, MSW, DKZ, RYJ, QHL, ZLC and XYC helped in experiments and drafted the manuscript. All authors read and approved the final manuscript.
